# The MmpL3 interactome reveals a complex crosstalk between cell envelope biosynthesis and cell elongation and division in mycobacteria

**DOI:** 10.1038/s41598-019-47159-8

**Published:** 2019-07-24

**Authors:** Juan Manuel Belardinelli, Casey M. Stevens, Wei Li, Yong Zi Tan, Victoria Jones, Filippo Mancia, Helen I. Zgurskaya, Mary Jackson

**Affiliations:** 1 Mycobacteria Research Laboratories, Department of Microbiology, Immunology and Pathology, Colorado State University, Fort Collins, CO 80523-1682 USA; 2 University of Oklahoma, Department of Chemistry and Biochemistry, 101 Stephenson Parkway, Norman, OK 73019 USA; 3 Department of Physiology and Cellular Biophysics, Columbia University, 1150 St. Nicholas Avenue, New York, NY 10032 USA

**Keywords:** Microbiology, Infectious diseases

## Abstract

Integral membrane transporters of the Mycobacterial Membrane Protein Large (MmpL) family and their interactome play important roles in the synthesis and export of mycobacterial outer membrane lipids. Despite the current interest in the mycolic acid transporter, MmpL3, from the perspective of drug discovery, the nature and biological significance of its interactome remain largely unknown. We here report on a genome-wide screening by two-hybrid system for MmpL3 binding partners. While a surprisingly low number of proteins involved in mycolic acid biosynthesis was found to interact with MmpL3, numerous enzymes and transporters participating in the biogenesis of peptidoglycan, arabinogalactan and lipoglycans, and the cell division regulatory protein, CrgA, were identified among the hits. Surface plasmon resonance and co-immunoprecipitation independently confirmed physical interactions for three proteins *in vitro* and/or *in vivo*. Results are in line with the focal localization of MmpL3 at the poles and septum of actively-growing bacilli where the synthesis of all major constituents of the cell wall core are known to occur, and are further suggestive of a role for MmpL3 in the coordination of new cell wall deposition during cell septation and elongation. This novel aspect of the physiology of MmpL3 may contribute to the extreme vulnerability and high therapeutic potential of this transporter.

## Introduction

**Figure 1:**
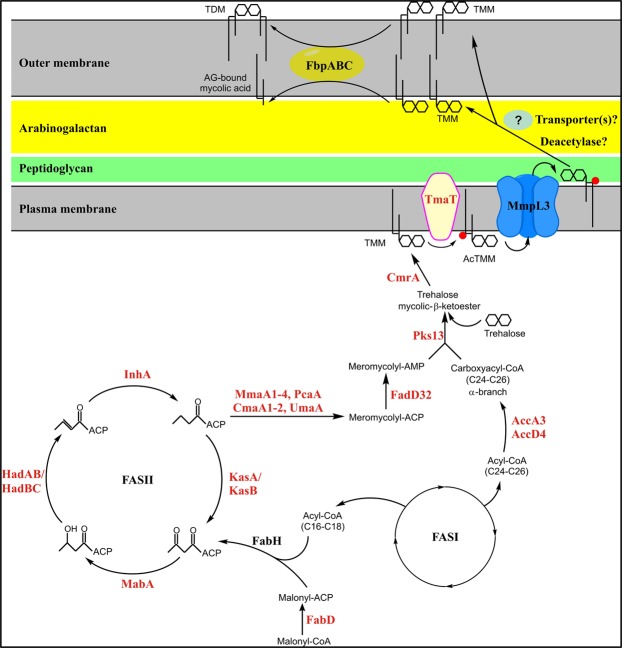
The *M*. *tuberculosis* mycolic acid biosynthetic pathway. The C48–C54 meromycolate chain is biosynthesized by FAS-II through the processive addition of multiple malonate units onto C16–C26 precursors generated by FAS-I. The initial substrates of FAS-II are thus medium length keto-acyl-ACP resulting from the condensation by the *M*. *tuberculosis* FabH protein of the acyl-CoA products of FAS-I with malonyl-ACP. After reduction by the β-keto-acyl-ACP reductase MabA, dehydration by the (3R)-hydroxyacyl dehydratases HadAB and HadBC, and reduction by the enoyl-CoA reductase InhA, either the β-ketoacyl-ACP synthase KasA or KasB catalyzes the condensation of the resulting product with malonyl-ACP units, thereby initiating the next round of elongation. The products of FAS-II may undergo further elongation and functional modifications of the meromycolic acid chain, catalyzed in part by *S*-adenosyl methionine-dependent methyltransferases (MmaA1, MmaA2, MmaA3, MmaA4, CmaA1, CmaA2, PcaA, UmaA), prior to the Pks13/FadD32-mediated condensation of the activated α-branch with the meromycolic acid chain to yield the full-size mycolic β-ketoester. Pks13 transfers mycolic β-ketoester to trehalose prior to reduction by the CmrA reductase yielding mature TMM. TmaT acetylates the mycolic acid in TMM prior to its export by MmpL3 and other potential components of a translocation machinery spanning the mycobacterial cell envelope. The mycolyltransferases encoded by *fbpA*, *fbpB* and *fbpC* catalyze the transfer of mycolic acids from TMM to arabinogalactan (AG), or to another TMM molecule generating TDM^69^. Proteins in red font were tested individually for interaction with MmpL3 by BATCH two-hybrid system.

Mycolic acids are essential building blocks of the outer membrane of all mycobacteria. The importance of drugs targeting their biosynthesis and export is illustrated by the therapeutic efficacy of such anti-mycobacterial agents as isoniazid, ethionamide, thiacetazone and isoxyl, and a number of recently discovered small molecule inhibitors reported to inhibit the integral membrane mycolic acid transporter, MmpL3^1,2^. Studies from our group and others have established that MmpL3, a member of the Resistance, Nodulation and Division (RND) superfamily, is required for the translocation of mycolic acids in the form of trehalose monomycolates (TMM) from the cytoplasm to the periplasm or outer membrane where this glycolipid can then serve as a mycolic acid donor for the enzymes catalyzing their transesterification to arabinogalactan or extracellular TMM yielding trehalose dimycolates (TDM) [Fig. 1]^3–5^.

Interestingly, multiple cell envelope-related biosynthetic pathways (e.g., sulfolipids, phthiocerol dimycocerosates, polyacyltrehaloses, glycopeptidolipids, siderophores, etc.) in mycobacteria and closely related Actinomycetes rely on related MmpL (*m*ycobacterial *m*embrane *p*roteins, *l*arge) proteins for the translocation of their products to their final extra-cytoplasmic location^6,7^. MmpL proteins typically interact with other enzymes and transporters of the same biosynthetic pathway and these physical interactions are required for efficient coupling of synthesis and export^6–8^. Although earlier work has begun to shed light on the nature and functional relevance of physical interactions between mycolic acid biosynthetic enzymes^9–12^, it has thus far only focused on known cytosolic enzymes and has not integrated any aspects of the translocation machinery. As a result, nothing is presently known of the spatial and temporal coupling of mycolic acid biosynthesis and export.

Given the challenge represented by the translocation of such high molecular weight, hydrophobic, constituents as mycolic acids across the different layers of the mycobacterial cell envelope, the existence of a molecular scaffold providing continuity between the cytoplasm, periplasm and the surface of mycobacteria also seems likely. This assumption is further supported by our single-particle electron microscopy studies showing that the length of MmpL3 (100 Å of which an estimated 35 Å extends in the periplasmic space) is not compatible with direct substrate delivery to the outer membrane (the thickness of the mycobacterial cell envelope is in the range of ∼35–40 nm)^13–15^. The first evidence of the existence of accessory proteins required for TMM export was obtained very recently using a co-purification strategy to identify proteins interacting with MmpL3 in *Mycobacterium smegmatis*. One plasma membrane-anchored protein named TtfA was shown to be required for TMM export whereas another protein co-eluting with MmpL3 was proposed to play a role in the stabilization of the MmpL3-TtfA complex under stress conditions^16^. Whether additional periplasmic adapters, outer membrane proteins and, perhaps, inner membrane transporters work with MmpL3 to bring TMM to the cell surface, similarly to the situation with prototypical HAE1 RND efflux pumps^17^ remains to be determined.

Another unique aspect of the biology of mycobacteria and other bacteria of the order Actinomycetales is the fact that insertion of newly synthesized peptidoglycan during elongation occurs at the cell poles rather than along the lateral cell wall as in other rod-shaped bacteria such as *Escherichia coli* and *Bacillus subtilis*^18,19^. Bacilli division and elongation without compromising the integrity of their existing multilayered cell wall implies spatiotemporal coordination of biosynthetic and export activities at the sites of septal and polar elongation, a process apparently facilitated in mycobacteria by the uneven distribution of the peptidoglycan, arabinogalactan, mycolic acid and phospho(glyco)lipid synthetic complexes in the inner membrane, and their enrichment in the polar and subpolar regions of actively elongating cells^20–24^. Despite these advances, much remains to be done in understanding the molecular mechanisms coordinating new cell wall deposition with cell septation/elongation, and characterizing the molecular interactions and dynamic features of the individual cell envelope biosynthetic pathways involved. The central involvement of MmpL3 in mycolic acid translocation, the localization of this transporter at the pole and septum of actively-growing cells^20,25^ where mycolic acid transfer to their outer membrane acceptors has been shown to occur^26–29^, and its ability to directly or indirectly interact with the elongation regulator Wag31 (aka DivIVA)^20^ led us to hypothesize that MmpL3 may be at the center of a protein scaffold coordinating new mycolic acid and, perhaps, other cell envelope constituents deposition during cell elongation and division.

The present interactome study was thus undertaken with three main aims. The first one was to determine whether MmpL3 acts as a scaffold for a multiprotein complex coupling mycolic acid synthesis and export. The second one was to identify putative additional components of the mycolic acid translocation machinery from *M*. *tuberculosis*. The third one was to begin delineating the other key events at the intersection of cell wall biogenesis, cell elongation and cell division in which MmpL3 may be involved. The results of these studies point to a complex crosstalk between mycolic acid, lipid and polysaccharide biosynthesis, and cell elongation and division in mycobacteria.

## Results

### Identification of MmpL3 binding partners using a bacterial two-hybrid system

We resorted to the *E*. *coli* adenylate cyclase two-hybrid (BACTH) system as our primary approach to identify *M*. *tuberculosis* proteins interacting with MmpL3. This system exploits the fact that the catalytic domain of the adenylate cyclase (CyaA) from *Bordetella pertussis* consists of two complementary fragments, T25 and T18, that are not active when physically separated but whose functional complementation when fused to interacting polypeptides results in cyclic AMP synthesis in an *E*. *coli cya* mutant^30^. cAMP produced by the reconstituted chimeric enzyme activates a β-galactosidase reporter allowing positive interactions to be easily identified on indicator agar media. Importantly, since this system relies on a signaling cascade that utilizes a diffusible regulatory molecule, it is suitable to detect interactions between cytoplasmic, as well as transmembrane and membrane-associated proteins^31,32^.

**Figure 2:**
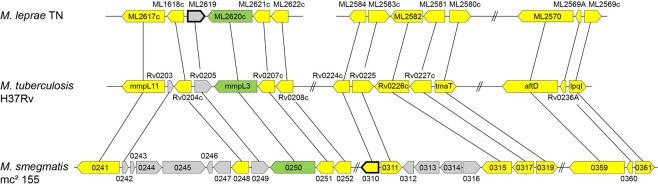
*mmpL3* genomic region in *M. tuberculosis*, *M. smegmatis* and *M. leprae*. Conserved genes are in yellow and were all tested for interaction with MmpL3 by BATCH two-hybrid system. Non-conserved genes (in grey) and pseudogenes (bold lines) were not tested. *mmpL3* and orthologs are in green.

MmpL3 fusion proteins harboring C-terminal or N-terminal T18 and T25 domains were generated as “baits” and systematically screened for pairwise interactions with four major sets of similarly fused proteins. Functional complementation efficiency between the T18 and T25 domains was quantified by measuring β-galactosidase activity. The first “unbiased” set, which was initially screened as pools of 96 baits against MmpL3, contained the entire *M*. *tuberculosis* Gateway® Clone Set from BEI Resources (NR-19274) consisting of 3,724 unique *M*. *tuberculosis* H37Rv and CDC1551 open reading frames (ORFs). Since the *M*. *tuberculosis* Gateway® Clone Set is missing a number of *M*. *tuberculosis* H37Rv ORFs, and because we also wanted to screen a number of target proteins individually against MmpL3, T18 and T25 C- and N-terminally-fused proteins were generated for the sets of test proteins whose description follows. One of these sets is represented on Fig. 1 and consists of all of the proteins known to be involved in mycolic acid biosynthesis. Given the fact that genes involved in the production and MmpL-dependent export of lipids tend to cluster in the genomes of mycobacteria^6^, another set of candidate interactors was further generated which consisted of all of the conserved genes clustering with *mmpL3* in *M*. *tuberculosis* and other mycobacterial genomes [Fig. 2]. Finally, fusions were prepared for candidate proteins that had either been proposed to interact with MmpL3 (e.g., Wag31)^20^ or were known to participate in lipid export in other MmpL-dependent pathways (e.g., Sap [Rv3821], the Sap-like protein Rv1517, and Rv0585c)^6,7^.

**Figure 3:**
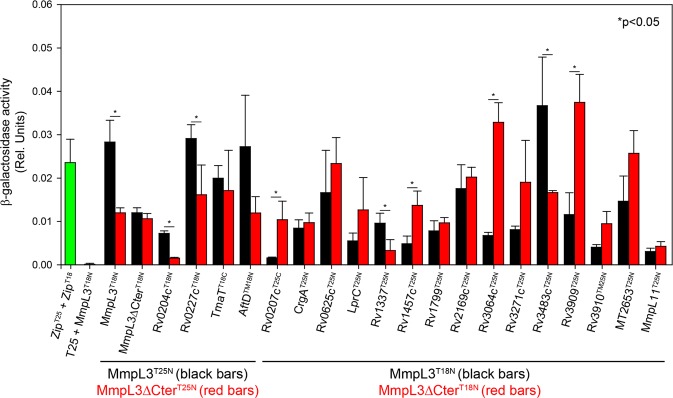
Quantification of *in vivo* interactions between full-size or C-terminal truncated MmpL3 and protein partners. MmpL3 (full-size) and MmpL3^1–744^ fusion proteins harboring C-terminal or N-terminal T18 and T25 domains were generated as “baits” and systematically compared for pairwise interactions with the binding partners listed in Table 1. Pairwise co-transformants developing a blue color in the BATCH screen were grown in LB broth, and the cultures processed for β-galactosidase activity as described in Methods. The values presented are the mean activities (relative units) ± standard error from measurements performed on three independent *E*. *coli* transformants.

*In toto*, this *in vivo* screen yielded 19 hits that reproducibly interacted with MmpL3 by BACTH two-hybrid system. These hits, their proposed localization in the cell and putative function are presented in Table 1. The results of the β-galactosidase activity assays are presented in Figs 3 and S1. Overall, the results which are detailed below highlighted a broad network of proteins participating in cell envelope biogenesis, cell elongation/division and as yet unknown functions with which MmpL3 appears to interact *in vivo*.

**Table 1:** BATCH analysis of the MmpL3 interactome.

Protein name	Proposed location	Proposed function	Conservation/essentiality	Ref.
Rv0011c (CrgA)	IM protein (2 TMS)	Divisome stabilization; PG assembly	mycobacteria-conserved Non-essential in *Msmg*; predicted non-essential in *Mtb*	^52, 53^
Rv0202c (MmpL11)	IM protein (12 TMS)	Translocation of monomeromycolyl diacylglycerol and mycolate wax ester	mycobacteria-conserved non-essential in *Mtb*	^35, 60^
Rv0204c	IM protein (8 TMS)	MprF-like (lysinylated phosphatidylglycerol flippase) protein	mycobacteria-conserved non-essential in *Mtb*	—
Rv0206c (MmpL3)	IM protein (12 TMS)	TMM export	mycobacteria-conserved essential for growth in *Mtb* and *Msmg*	^4, 56^
Rv0207c	cytoplasm	unknown	mycobacteria-conserved non-essential in *Mtb*	—
Rv0227c	IM protein (1 or 2 TMS)	LM/LAM elongation in *Cgl*	mycobacteria-conserved essential in *Msmg*; predicted essential in *Mtb*	^40^
Rv0228 (TmaT)	IM protein (9 TMS)	TMM acetylation	mycobacteria-conserved essential in *Msmg*; predicted essential in *Mtb*	^15, 33^
Rv0236c (AftD)	IM protein (9 TMS)	AG and LAM biosynthesis (arabinosyltransferase)	mycobacteria-conserved essential in *Msmg*; predicted essential in *Mtb*	^41, 42^
Rv0625c	IM protein (5 TMS)	unknown	absent from *M*. *leprae;* clusters with AG and mycolic acid biosynthetic genes predicted non-essential in *Mtb*	—
Rv1275 (LprC)	periplasm (lipoprotein)	unknown	mycobacteria-conserved clusters with other transport genes predicted non-essential in *Mtb*	—
Rv1337	IM protein (6 TMS)	unknown (rhomboid protease 2)	mycobacteria-conserved clusters with *murI* (PG synthesis) non-essential in *Mtb* and *Msmg*	^45, 46^
Rv1457c	IM protein (6 TMS)	ABC-transporter throught to be involved in LM/LAM biosynthesis	mycobacteria-conserved predicted essential in *Mtb*	^47^
Rv1799 (LppT)	periplasm (lipoprotein)	unknown	Restricted to *Mtb* and *M*. *bovis* non-essential in *Mtb*	—
Rv2169c	IM protein (2 TMS)	unknown	mycobacteria-conserved; clusters with cell division regulators and LM/LAM biosynthetic genes non-essential in *Mtb*	—
Rv3064c	IM protein (4 TMS)	unknown	absent from *M*. *leprae* non-essential in *Mtb*	—
Rv3271c	IM protein (4 to 6 TMS)	unknown	not conserved in mycobacteria predicted non-essential in *Mtb*	—
Rv3483c	exported protein	unknown	not conserved in mycobacteria; adja-cent to cell wall ligase gene *cpsA2* non-essential in *Mtb*	^51^
Rv3909	putative outer membrane	unknown	mycobacteria-conserved adjacent to *mviN* predicted essential in *Mtb*	—
Rv3910 (MviN)	IM protein (15 TMS)	proposed lipid II flippase (PG biosynthesis)	mycobacteria-conserved essential in *Msmg*; predicted essential in *Mtb*	^43^
MT2653	cytoplasm	unknown	CDC1551-specific; not in H37Rv	—

Fusions proteins made of MmpL3 and other *M*. *tuberculosis* proteins harboring C-terminal or N-terminal T18 and T25 domains were transformed in *E*. *coli* BTH101 cells in different combinations, and the transformants grown at 30 °C for 48 hours on LB/X-Gal/IPTG agar. The positive control consisted of the fusion proteins combination pKT25-Zip + pUT18C-Zip (provided with the BACTH system kit) and the negative control, of the empty plasmids pKT25 + pUT18. Individual colonies were grown in LB broth, and the cultures processed for β-galactosidase activity as described in Methods and Fig. 3. All co-transformations were repeated at least three times to confirm pairwise interactions. IM protein, integral membrane protein; TMS, transmembrane segment; PG, peptidoglycan; AG, arabinogalactan; *Msmg*, *M*. *smegmatis*; *Mtb*, *M*. *tuberculosis*.

### Interaction of MmpL3 with components of the mycolic acid biosynthetic machinery and products of syntenic genes

**Figure 4:**
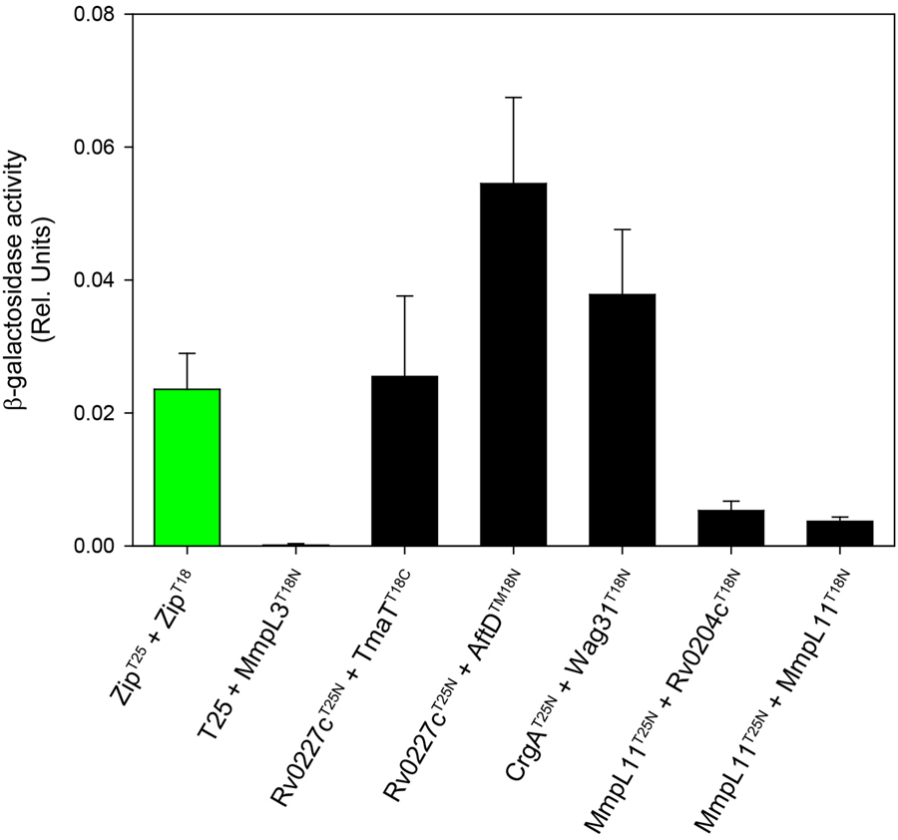
Quantification of *in vivo* interactions between MmpL3 binding partners. CrgA, Wag31, TmaT, AftD, Rv0227c, Rv0204c and MmpL11 fusion proteins harboring C-terminal or N-terminal T18 and T25 domains were generated as “baits” and tested for pairwise interactions with other MmpL3 binding partners. Pairwise co-transformants developing a blue color in the BATCH screen were grown in LB broth, and the cultures processed for β-galactosidase activity as in Fig. 3.

Consistent with our earlier finding that the full-size MmpL3 protein functions as a homotrimer^15^, MmpL3 interacted with itself [Fig. 3]. Of the known mycolic acid biosynthetic enzymes that might have interacted with MmpL3 in the cytoplasm or in the membrane itself, none of the enzymes involved in the synthesis of meromycolate precursors (e.g., FAS-II enzymes), functionalization of the meromycolate chain (*S*-adenosyl-methionine methyltransferases) or final assembling of the meromycolate with the alpha-branch (Pks13, FadD32) [Fig. 1] were identified as MmpL3 binding partners under the assay conditions used herein [Fig. S1]. In fact, the only interactor identified within this pathway was TmaT, an acyltransferase responsible for the acetylation of mycolic acids in TMM whose activity appears to be a requisite for TMM export and cell viability in mycobacteria^15,33^. TmaT is thought to catalyze the last modification of TMM prior to export by MmpL3. *tmaT* clusters with *mmpL3* in the same genomic region, as do a number of other binding partners identified in this screen (MmpL11; Rv0204c; Rv0207c; Rv0227c; AftD). *Rv0207c*, a *Mycobacterium*-conserved gene of unknown function, maps adjacent to *mmpL3*. *Rv0204c* encodes a potential inner membrane transporter with homology to MprF translocases. Multiple Peptide Resistance Factor (MprF) proteins represent a highly conserved protein family responsible for the synthesis and translocation of aminoacyl phospholipids in a variety of bacteria^34^. Rv0204c, however, is unusual in that it is devoid of the biosynthetic domain of MprF proteins and only harbors the translocase domain. MmpL11 (Rv0202c) is involved in the translocation of the mycolic acid-containing lipids, monomeromycolyl diacylglycerol and mycolate wax ester^35^ and, together with MmpL3, was proposed to participate in heme-iron uptake on the basis of the ability of the periplasmic domains of these two RND transporters to bind heme^36,37^. While the two transporters only showed weak interaction between them [Fig. 3], they both were identified as binding partners of the MprF-like translocase Rv0204c [Figs 3 and 4] suggestive of some form of coupling of the two MmpL-dependent pathways. Although *Rv0204c* is not required for growth, the disruption of this gene has been reported to attenuate *M*. *tuberculosis* virulence^38,39^. Rv0227c was recently shown to serve a critical, albeit incompletely defined, function in the elongation of lipomannan (LM) and lipoarabinomannan (LAM) in *Corynebacterium glutamicum*^40^, while AftD (Rv0236c) plays an essential role in the arabinosylation of LAM and arabinogalactan in mycobacteria^41,42^.

### Lack of periplasmic interactions between MmpL3 and the FbpA, FbpB and FbpC mycolyltransferases

Three mycolyltransferases in *M*. *tuberculosis* - FbpA (Rv3804c), FbpB (Rv1886c) and FbpC (Rv0129c) - catalyze, on the periplasmic side of the plasma membrane, the transfer of mycolic acids from TMM to their cell envelope acceptors [Fig. 1]. Since the periplasmic location of these enzymes precluded the use of the BACTH system to detect potential interactions with MmpL3, Surface Plasmon Resonance (SPR) was used instead. To this end, the MmpL3 protein from *M*. *tuberculosis* was produced in a *M*. *smegmatis* knock-out mutant devoid of its endogenous *mmpL3* gene and purified using a combination of nickel affinity and ion-exchange chromatography [Fig. S2]. The purified MmpL3 protein was then immobilized onto the surface of CM5 chips as described under Methods, and increasing concentrations of the analyte proteins were injected over the surface. SPR assays with the purified native FbpA, FbpB and FbpC proteins failed to reveal any interaction with MmpL3 [Fig. S3]. Similarly, testing of purified Pks13 protein by SPR confirmed the lack of interaction between MmpL3 and this polyketide synthase [Fig. S3].

### MmpL3 binding partners with other known or proposed functions in cell envelope biogenesis

Interestingly, the BACTH screening identified a number of other MmpL3-binding partners outside the *mmpL3* cluster whose demonstrated or proposed function, or clustering with cell envelope-related genes in the *M*. *tuberculosis* genome, are suggestive of their participation in cell envelope biogenesis. Foremost among these are three highly conserved proteins involved, or thought to be involved, in peptidoglycan synthesis and export: Rv1337, Rv3909 and Rv3910 (MviN) [Fig. 3]. MviN is required for peptidoglycan precursor export or polymerization; by analogy with Gram-negative MviN proteins, it was proposed to act as a lipid II flippase^43^. Rv3909 was predicted by bioinformatics approaches to be an outer membrane protein^44^ although experimental proof for this assumption is currently lacking. The organization of the *Rv3909* and *mviN* loci in the *M*. *tuberculosis* genome suggests that the two genes are probably co-transcribed. Rv1337 is a conserved rhomboid-like protein of unknown function whose encoding gene clusters with the essential peptidoglycan biosynthetic gene *murI* in all mycobacterial genomes^45^. Disruption of this gene in *M*. *smegmatis* was found to alter the ability of bacilli to form biofilms and their susceptibility to hydrophobic antibiotics suggestive of changes in cell surface properties and cell envelope permeability^46^.

Other interactors potentially involved in cell envelope synthesis include Rv0625c, a protein of unknown function whose encoding gene clusters with a number of arabinogalactan (*galTa*, *galTb*) and mycolic acid biosynthetic genes (*hadA*, *hadB*, *hadC*). Rv1457c, an inner membrane component of an ABC-transporter whose genetic association with a mannosyltransferase involved in LM/LAM synthesis (Rv1459c/MptB) is suggestive of its involvement in the export of lipoglycans^47^. Rv2169c maps in a region of the *M*. *tuberculosis* genome rich in recently characterized cell division regulator genes (*Rv2151*, *Rv2164*)^48^ as well as peptidoglycan, LM, LAM and phospholipid biosynthetic genes (*Rv2163c*/*pbpB; Rv2174/mptA*, *Rv2181*, *Rv2182c*)^49^. *Rv3483c* is adjacent to the cell wall ligase gene *cpsA2* (*Rv3484*) involved in the covalent attachment of arabinogalactan with peptidoglycan^50^, and the ortholog of this protein in *M*. *marinum* is apparently secreted by the ESX-1 secretion system^51^. All of these proteins and other interactors of as yet unknown function presented in Table 1 (Rv3064c, Rv3271c, LprC, LppT) are predicted to be periplasmic, integral membrane or outer membrane proteins and may participate in the export of TMM or that of other as yet unknown cell envelope constituents. We note that the finding of lipoproteins and other potentially exported proteins in our screen was unexpected as the BACTH system is not designed to reveal extra-cytoplasmic interactions. These results may indicate false-positives. Alternatively, the improper export or insertion in the plasma membrane of some of the test proteins and MmpL3 fusions produced in *E*. *coli* may inadvertently have led extra-cytoplasmic interactors of MmpL3 to interact with periplasmic regions of this transporter in the cytoplasm of *E*. *coli*.

### Interaction of MmpL3 with proteins involved in cell elongation and septation

An earlier interactome study identified MmpL3 among proteins co-immunoprecipitating with Wag31^20^, an elongation specific regulatory factor involved in the coordination of cell envelope biogenesis at the older, faster growing, pole of mycobacteria^21^. While the BACTH assay failed to reveal any direct interaction between MmpL3 and Wag31 (data not shown), the screening of the *M*. *tuberculosis* genomic “prey” library from BEI Resources identified CrgA as an MmpL3 interactor [Fig. 3]. CrgA is a cell division regulatory protein that facilitates septum formation and ensures proper septal and polar peptidoglycan assembly by influencing the localization of penicillin-binding proteins and Wag31^52,53^. The identification of CrgA as a protein interacting with MmpL3 unveils a plausible mechanism through which MmpL3 may be recruited to the septum to drive mycolic acid deposition at this site during cell division^25,29^. Given that CrgA interacts with Wag31 [Fig. 4], it is possible that CrgA further contributes to the relocalization of MmpL3 to the old poles during cell elongation^21,25,29^.

### Validation of MmpL3 interactions with CrgA, Rv0207c and AftD by surface plasmon resonance

**Figure 5:**
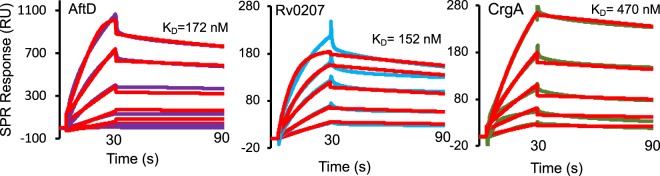
Quantification of *in vitro* interactions between MmpL3 and binding partners by surface plasmon resonance. SPR was used to analyze kinetics of the purified MmpL3 interaction with various proteins. Binding sensorgrams were collected by injecting two-fold increasing concentrations ranging from 0.6 μM up to 10 μM of AftD (violet), Rv0207c (blue) and CrgA (green). Binding curves were fitted globally into a simple 1:1 model (red lines) and the dissociations constants are shown for each protein. No specific binding was detected for the Ag85 complex, Pks13, MviN and LprC [see Fig. S3].

We next turned to an independent *in vitro* protein-protein interaction approach, namely SPR, to validate the results of some of our BACTH assays with a subset of proteins selected to encompass the variety of functions (mycolic acid biosynthesis, peptidoglycan arabinogalactan and lipoarabinomannan synthesis, cell elongation and division) and subcellular localizations (inner membrane, periplasm, cytoplasm) uncovered by the two-hybrid system screening. To this end, the MviN, AftD, LprC, CrgA and Rv0207c proteins were produced in *E*. *coli* and purified by a combination of ion exchange and metal affinity chromatographies as described under Methods [Fig. S2]. Attempts to produce and purify TmaT unfortunately failed, both in *E*. *coli* and *M*. *smegmatis*. The purified MmpL3 was then captured onto the surface of CM5 chips and interactions with the candidate proteins were analyzed as described for FbpA, FbpB, FbpC and Pks13, by injecting over the surface increasing concentrations of MviN, AftD, LprC, CrgA and Rv0207c. The results, which are presented in Fig. 5, indicated that CrgA, Rv0207c and AftD, but not LprC or MviN [Fig. S3], bind specifically and with high affinities to the immobilized MmpL3. The collected CrgA, AftD and Rv0207c sensorgrams were fit globally into different kinetic models to analyze the mechanism and affinity of the interactions. The goodness of obtained fits was comparable for different models, and a simple 1:1 binding model was used further to compare the affinities of the observed protein-protein interactions. For all three proteins, the dissociation constants (K_D_) for the interaction with MmpL3 were found to be in the mid nanomolar range [Fig. 5 and Table S1]. Thus, MmpL3 interacts directly and with comparable affinities with CrgA, AftD and Rv0207c. Failure to detect an interaction between LprC and MmpL3 supports our earlier assumption that possibly several, if not all, of the interactions revealed by BACTH between MmpL3 and exported proteins may in fact be false positives. The lack of detectable interaction between MviN and MmpL3 in the SPR assay may likewise either indicate a false positive or result from the improper folding or oligomerization of the recombinant form of MviN produced in *E*. *coli*.

### MmpL3 interaction with CrgA in intact *M. smegmatis* cells

**Figure 6:**
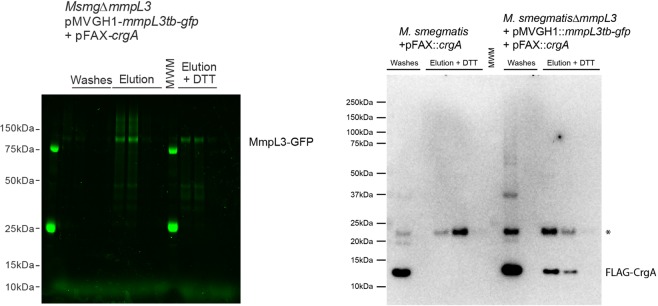
CrgA interacts with MmpL3 in intact mycobacterial cells. *Left*: In-gel fluorescence of DSP-treated, detergent-solubilized, MmpL3tb-protein complexes prepared from *Msmg*Δ*mmpL3*/pMVGH1-*mmpL3tb-gfp* + pFAX-*crgA* cells reveals the presence of high molecular weight MmpL3-GFP protein complexes in the elution fractions that are reduced upon addition of DTT. The expected size of MmpL3-GFP is ~126 KDa. *Right*: Immunoblot analysis of DSP-treated, detergent-solubilized, MmpL3tb-protein complexes prepared from *Msmg* + pFAX-*crgA* and *Msmg*Δ*mmpL3*/pMVGH1-*mmpL3tb-gfp* + pFAX-*crgA* cells. The immunoblot shows the presence of CrgA-FLAG in the elution fractions from *Msmg*Δ*mmpL3*/pMVGH1-*mmpL3tb-gfp* + pFAX-*crgA* cells but not in those from cells devoid of *mmpL3tb-gfp* expression plasmid. The expected size of CrgA-FLAG is ~12 KDa. *Denotes a non-specific *M*. *smegmatis* protein reacting with the anti-FLAG antibody. The full-length gel and blots are shown. Co-affinity purifications were performed twice on independent culture batches with the same results.

To determine whether MmpL3 interacted with CrgA *in vivo* in the context of intact *M*. *smegmatis* cells, we finally resorted to co-immunoprecipitation. To this end, a recombinant CrgA protein N-terminally-tagged with a FLAG epitope was expressed in the background of *Msmg*Δ*mmpL3*/pMVGH1-*mmpL3tb-gfp* from the integrative plasmid pFAX-*crgA*. Intact *M*. *smegmatis* cells co-expressing CrgA-FLAG and MmpL3tb-GFP tagged with hexahistidine at the C-terminal end were treated with the cross-linking agent dithiobis[succinimidyl propionate] (DSP) to cross-link protein complexes before breaking the cells. MmpL3tb was next purified from these cells by Ni-NTA affinity chromatography, and the presence of CrgA-FLAG in MmpL3-containing elution fractions was investigated by immunoblot using antibodies to the FLAG epitope. The elution fractions from *M*. *smegmatis* cells missing either the expression plasmid for CrgA-FLAG or pMVGH1-*mmpL3tb-gfp* were used as negative controls. As shown in Fig. 6, CrgA-FLAG co-eluted with MmpL3tb-GFP, yielding a FLAG-positive signal of the expected size for CrgA-FLAG (~12 kDa) upon reduction of protein complexes with dithiothreitol. In contrast, CrgA-FLAG was missing in the pulldown samples similarly prepared from *M*. *smegmatis* cells missing either pFAX-*crgA* or pMVGH1-*mmpL3tb-gfp* [Figs 6 and S4]. Thus, CrgA appears to interact with MmpL3tb in intact mycobacterial cells. Either due to low abundance or to untoward exposure of the FLAG epitope within protein complexes, CrgA-FLAG was not directly detected in the high molecular weight MmpL3-GFP complexes and was only visible upon DTT treatment [Fig. 6]. Furthermore, no CrgA was detected in the elution fraction when the crosslinker was omitted from the cell preparation (data not shown).

### Impact of the C-terminal cytoplasmic domain of MmpL3 on protein interactions

**Figure 7:**
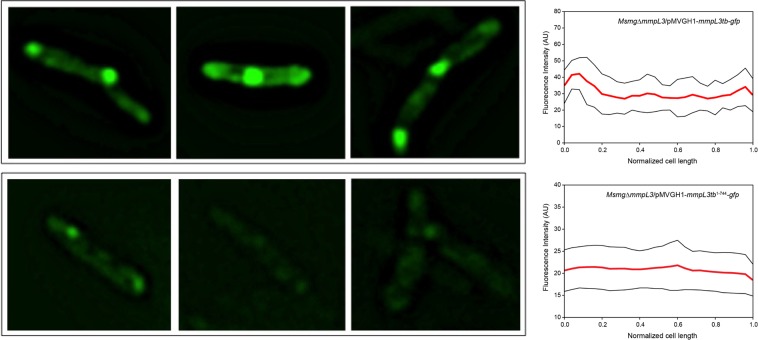
Loss of polar localization of MmpL3 upon truncation of the C-terminal domain. Representative fluorescence images of *Msmg*Δ*mmpL3*/pMVGH1-*mmpL3tb-gfp* (top panels) and *Msmg*Δ*mmpL3*/pMVGH1-*mmpL3tb*^1–744^*-gfp* (lower panels) showing the focal localization of full-size MmpL3tb-GFP at the old poles and septa of actively dividing bacilli, and dimmer and more diffuse fluorescent signal corresponding to the truncated form of the transporter. The fluorescence intensity of 50 bacilli from each strain (red line) in one representative experiment, plus or minus standard deviation (black lines), was scored and plotted against normalized cell length (right panels).

The ability of MmpL3 to accumulate at the old pole and septa of actively growing cells was shown to be dependent on a region of the transporter located between the end of its tenth transmembrane segment (TMS) and the C-terminus^25^. To more precisely delineate the region of MmpL3 required for its dynamic localization in growing bacilli, *M*. *smegmatis* recombinant strains were generated of which the endogenous *mmpL3* gene was entirely deleted and replaced by one of two different, ectopically expressed, rescue copies of the *mmpL3* gene from *M*. *tuberculosis*. One rescue copy corresponded to the full-size *mmpL3* gene fused at its 3′ end to *gfp* (yielding *Msmg*Δ*mmpL3*/pMVGH1-*mmpL3tb-gfp*). The second rescue copy corresponded to a 3′-truncated form of the gene encoding the first 744 amino acid residues of the protein C-terminally fused to GFP (yielding *Msmg*Δ*mmpL3*/pMVGH1-*mmpL3tb*^1–744^*-gfp*). Compared to the truncated form of MmpL3 studied by Carel *et al*.^25^, MmpL3tb^1–744^-GFP harbors the last two transmembrane domains of the transporter (TMS11 and TMS12) and is thus only devoid of the cytosolic C-terminal domain of the protein. Consistent with our earlier findings^15^, the *Msmg*Δ*mmpL3* strain rescued with *mmpL3tb*^1–744^*-gfp* was viable indicating that MmpL3tb^1–744^-GFP was competent at exporting mycolic acids in actively replicating bacilli. Observation of *Msmg*Δ*mmpL3*/pMVGH1-*mmpL3tb-gfp* bacilli by fluorescence microscopy revealed a preferential labeling of the poles and septa [Fig. 7, top panels]. Thus, despite expressing the *M*. *tuberculosis* ortholog of MmpL3, *M*. *smegmatis* was able to successfully recruit the transporter to its poles. Fluorescence in *Msmg*Δ*mmpL3*/pMVGH1-*mmpL3tb*^1–744^*-gfp* bacilli, in contrast, was dimmer and more diffuse, with reduced accumulation of MmpL3 at the poles and septa, and GFP signals being more evenly distributed along the lateral wall of the bacilli [Fig. 7, lower panels]. In line with Carel *et al*.’s observations^25^, the polar localization of MmpL3 was thus reliant on the C-terminal region of the protein, which may now be narrowed down to the last 200 cytoplasmic residues of the transporter.

In an attempt to begin elucidating the molecular mechanisms accounting for the decreased polar localization of MmpL3tb^1–744^-GFP in *M*. *smegmatis*, all the MmpL3 binding partners identified in our two-hybrid system screen were re-tested for interaction with MmpL3tb^1–744^ using the BACTH assay. The results, which are described on Fig. 3, revealed different patterns of behavior depending on the protein. While some interactions increased in intensity (e.g., Rv0207c, Rv1457c, Rv3064c, Rv3909), Rv0204c, Rv0227c, Rv1337 and Rv3483c appeared, on the contrary, to interact less strongly with MmpL3tb^1–744^. Changes in interactions with other proteins were not statistically significant. Of note was the decreased interaction between MmpL3tb and MmpL3tb^1–744^ monomers as well as between MmpL3tb^1–744^ and MmpL3tb^1–744^ monomers [Fig. 3] suggesting that the C-terminal end of MmpL3 plays a role in the stable oligomerization of the transporter. This finding may explain why C-terminally truncated forms of MmpL3 were recently reported to crystallize as monomers^54,55^ rather than as trimers^15^. Interestingly, no significant differences in binding were detected between CrgA and MmpL3tb full-size, and CrgA and MmpL3tb^1–744^ [Fig. 3] indicating that the MmpL3/CrgA interaction is not through the C-terminal domain of the transporter. Since the C-terminal domain of MmpL3 is critical to its polar localization, this result suggests that proteins other than CrgA are responsible for the recruitment of the transporter at the old poles and septa. Alternatively or in addition, the decreased interaction between MmpL3 monomers and failure of MmpL3 to properly oligomerize following the truncation of its C-terminal domain may contribute to the more diffuse fluorescence signal in *M*. *smegmatis* expressing the truncated MmpL3tb^1–744^-GFP than those expressing the full-size MmpL3-GFP.

## Discussion

**Figure 8:**
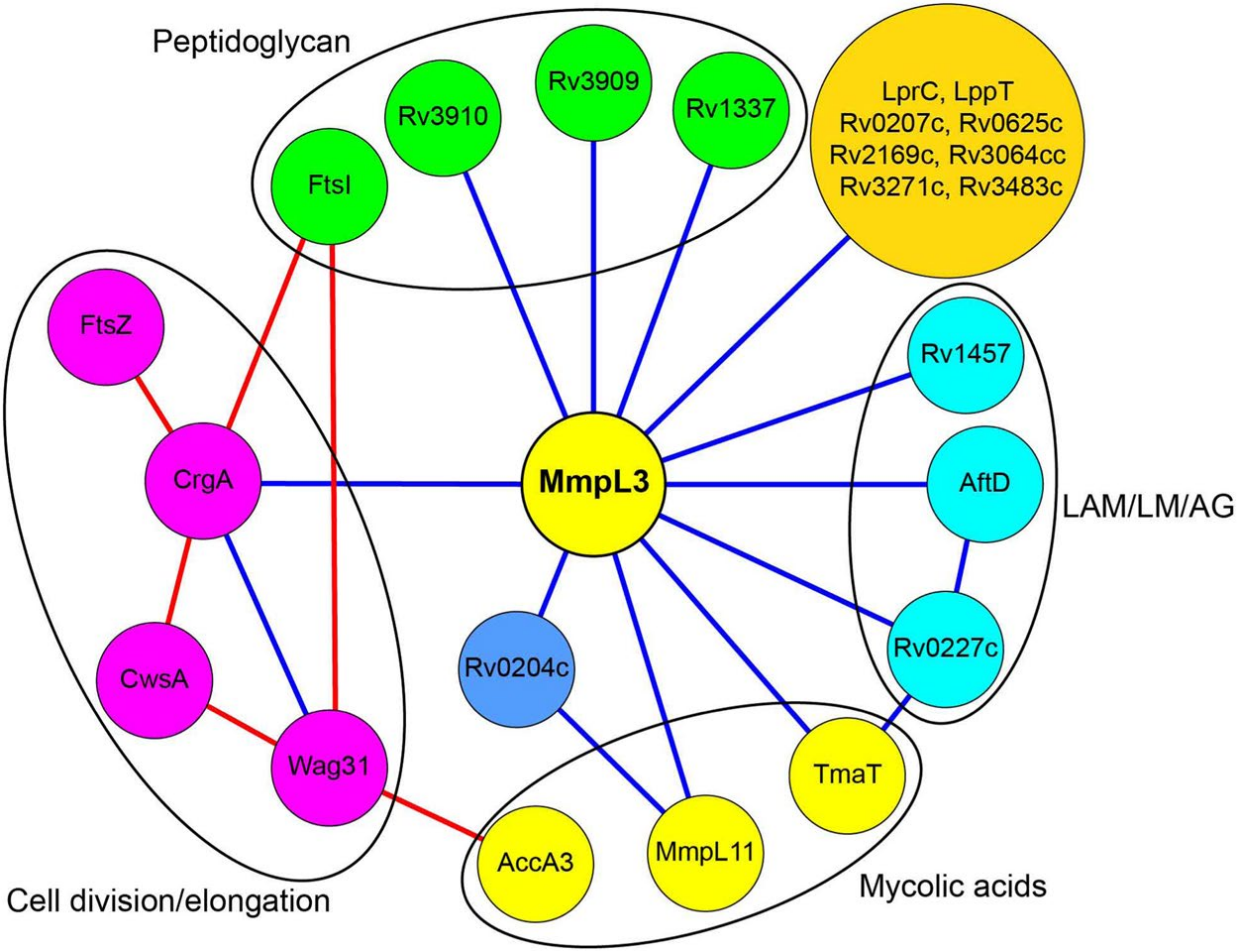
Network of *M*. *tuberculosis* proteins found to interact with MmpL3. Blue lines indicate protein interactions established in the context of the present study. Protein-protein interactions established in the context of previous studies are connected by red lines^20,52,53^. The functional groups to which the proteins belong or are thought to belong are color-coded. Proteins in yellow circles are related to mycolic acid metabolism. Proteins in the orange circle have not yet been assigned a function. LAM, lipoarabinomannan; LM, lipomannan; AG, arabinogalactan.

Our screen for proteins interacting with MmpL3 identified an unexpectedly low number of hits among enzymes participating in the biosynthesis of mycolic acids. The acetyltransferase TmaT was in fact the only hit in this pathway. Under the conditions of our assays, neither Pks13 nor the mycolyltransferases of the antigen 85 complex that catalyze sequentially close biosynthetic steps upstream and downstream of MmpL3, respectively [Fig. 1], were found to be binding partners. MmpL3, however, was revealed by our screen as a likely protein scaffold for a number of other critically important cell envelope-related biosynthetic machineries including those involved in the assembling and export of peptidoglycan (MviN, CrgA, and possibly Rv3909 and Rv1337), LM, LAM and arabinogalactan (AftD, Rv0227c, Rv1457c), monomeromycolyl diacylglycerol and mycolate wax ester (MmpL11), and perhaps aminoacyl phospholipids (Rv0204c/MprF) [Fig. 8]. Of these, CrgA, Rv0207c, and AftD belonging to various independent functional clusters represented on Fig. 8 were validated by biophysical and/or biochemical methods to physically interact with MmpL3. The existence of a tight network of physically-interacting cell envelope-related pathways was further supported by the detection of interactions between MmpL3 binding partners, e.g., MmpL11/Rv0204c; Rv0227c/TmaT and Rv0227c/AftD [Fig. 4]. Importantly, in addition to interacting indirectly with Wag31^20^, MmpL3 was also found to interact with the major cell division regulatory protein CrgA pointing to a plausible mechanism through which CrgA, either directly or through its interaction with Wag31 [Fig. 4], may participate in the relocalization of MmpL3 to the septal and polar regions of actively growing cells to promote mycolic acid deposition at these specific sites^21,24,25,29^. That CrgA is not the only protein contributing to the accumulation of MmpL3 at these specific locations, however, was suggested by the lack of effect of truncating the C-terminal end of the transporter on its ability to interact with CrgA in the BACTH assay, yet, the loss of clear polar localization of the truncated form of MmpL3 in *M*. *smegmatis* cells. Collectively, the results presented herein are thus not only suggestive of the existence of a broad connectivity between cell envelope biosynthetic machineries in mycobacteria but also indicate that MmpL3 may serve as an anchor in the coordination of new cell wall deposition with cell elongation and division at the old and septal poles. It is possible that this additional function of MmpL3 contributes to the extreme vulnerability of this therapeutic target^56,57^.

Previous studies have reported on the pleiotropic phenotypic effects of silencing or disrupting genes involved in essential mycobacterial cell envelope-related pathways without conclusively determining whether these effects were attributable to compensatory mechanisms, perturbation of the activity of other membrane enzymes and transporters, overall changes in cell envelope permeability, or other mechanisms. For instance the ABC transporter encoded by *Rv1458c-Rv1456c* was originally proposed to be involved in the translocation of short-chain corynomycolic acids on the basis of the phenotypic characterization of *Corynebacterium matruchotii* transposon mutants harboring insertions in this transporter^58^. The transcriptional coupling of this transporter with a mannosyltransferase responsible for the elongation of LM and LAM subsequently led Mishra *et al*.^47^ to propose that the altered mycolic acid profile of the *C*. *matruchotii* mutants was probably an indirect effect resulting from the loss of mature lipoglycans in the cells. Recently, the high-resolution lipidomics analysis of a *C*. *glutamicum tmaT* knock-out mutant revealed an unexpected accumulation of triglycerides in the inner membrane of the mutant that accompanied a decrease in phosphatidylglycerol, alanylated-phosphatidylglycerol and alanylated diacylglycerol^59^. Likewise, disruption of *mmpL11* in *M*. *tuberculosis* caused a decrease in cardiolipin content concomitant with alterations in triglyceride levels^60,61^. Finally, *mmpL3* silencing in *M*. *tuberculosis* was shown to lead to the up- or down-regulation of 70 genes among which genes encoding a variety of transporters and others involved in mycolic acid and lipid biosynthesis^57^. The results of our interactome studies strongly suggest that some of the above observations might in fact be the result of perturbations in protein-protein interactions caused by the gene knock-outs simultaneously affecting multiple biosynthetic processes.

It is noteworthy that although several MmpL proteins involved in the export of (glyco)lipids and siderophores in mycobacteria have been found to be genetically and functionally associated with membrane proteins facilitating substrate export (e.g., the ABC-transporter DrrABC, Mycobacteria Membrane Protein Small [MmpS] and small integral membrane proteins of the Gap/Sap family)^6,7^, no obvious homologs of these proteins are encoded by the genomic region surrounding *mmpL3* and no such protein was identified within the MmpL3 interactome reported herein. Our BACTH screen, however, identified a number of integral membrane, lipoproteins and other exported proteins of unknown function (Rv0204c, Rv0227c, Rv0625c, Rv1337, Rv2169c, Rv3064c, Rv3271c, Rv3483, LprC, LppT), some of which may facilitate the export of TMM or other cell envelope constituents. Lipoproteins (other than LprC which did not interact with MmpL3 by SPR), if confirmed to interact with MmpL3, are of particular interest in light of the previous involvement of some of them in the periplasmic translocation of triglycerides, polyketide-derived lipids, glycolipids and lipoglycans^62–65^. More studies are required to confirm these potential transporters as binding partners of MmpL3 and to determine the functional significance of these interactions, both from the perspective of mycobacterial physiology and susceptibility to MmpL3 inhibitors.

## Methods

### Bacterial strains and growth conditions

*Escherichia coli* DH5α and XL1-Blue, the strains used for cloning purposes, were grown in LB broth and LB agar. *M*. *smegmatis* mc^2^ 155 was grown in Middlebrook 7H9-ADC medium supplemented with 0.2% glycerol and 0.05% Tween 80 or on 7H11-ADC agar supplemented with 0.2% Glycerol. For *E*. *coli*, kanamycin, ampicillin, spectinomycin and apramycin were used at 50, 100, 50 and 50 μg/mL, respectively. For *M*. *smegmatis*, kanamycin, hygromycin and apramycin were used at 25, 50 and 25 μg/mL, respectively.

### Two-hybrid system

Bacterial two-hybrid system experiments were conducted using the BACTH (Bacterial Adenylate Cyclase Two-Hybrid) System kit (Euromedex, France). *M*. *tuberculosis mmpL3* and target genes of interest were PCR-amplified and cloned into plasmids pKT25, pKNT25, pUT18 and pUT18C. Genes encoding membrane proteins with known or predicted periplasmic N-terminal ends (*aftD*, *mviN*) were also cloned in pUTM18C to redirect the T18 domain to the cytoplasm^66^. Target genes present in the *Mycobacterium tuberculosis* Gateway® Clone Set (BEI Resources) were directly subcloned into the Gateway®-compatible plasmids pST25-DEST, pSNT25-DEST or pUT18C-DEST^66^ using the Gateway® LR Clonase® enzyme (ThermoFisher) as recommended by the manufacturer. Approximately 10 ng of plasmid pairs in different combinations were used to co-transform BTH101 cells and transformants selected on LB agar containing appropriate antibiotics, 0.5 mM IPTG and 40 μg/mL X-Gal. Plates were incubated at 30 °C for 48 h prior to visual inspection for blue colonies indicative of protein interactions.

For the screening of a genomic library of *M*. *tuberculosis* open reading frames against *mmpL3*, we used the *M*. *tuberculosis* Gateway® Clone Set from BEI Resources (NR-19274) consisting of 3,724 unique *M*. *tuberculosis* H37Rv and CDC1551 ORFs cloned in vector pDONR™221 in *E*. *coli* DH10B-T1 cells. The forty-two 96-well plates carrying these clones were re-grown in LB-kanamycin medium after which the 96-wells from each plate were combined and the plasmids extracted, thereby yielding 42 pools of 96 plasmids. The *M*. *tuberculosis* ORFs from each plasmid pool were then subcloned into pST25-DEST and pSNT25-DEST using the Gateway® LR Clonase® enzyme as described above. LR Clonase reactions were next used to transform BTH101 competent cells harboring either pUT18::mmpL3 or pUT18C::mmpL3, and the transformation mixtures plated on five to ten LB plates containing ampicillin, spectinomycin, IPTG and X-Gal in order to select for blue colonies indicative of protein interactions. Plasmids purified from blue colonies were confirmed for positive interaction by co-transformation with empty pUT18 or pUT18C plasmids (negative controls) or plasmids harboring *mmpL3-T18* fusions (positive controls), and finally sequenced to identify the genes yielding positive interactions with *mmpL3*.

All pairwise co-transformants developing a blue color in the BATCH screen were grown in LB broth, and the cultures processed for β-galactosidase activity as described previously^67^. Briefly, 3 independent colonies per pairwise co-transformation were picked and grown overnight in 96-well plates containing LB medium supplemented with antibiotics and 0.5 mM IPTG. 50 μl of each culture were used to determine OD_600nm_ while another 200 μl was transferred into 96-well propylene blocks containing 800 μl of Z buffer (60 mM Na_2_HPO_4_, 40 mM NaH_2_PO_4_, 10 mM KCl, 1 mM MgSO_4_ and 50 mM β-mercaptoethanol). After adding 20 μl of freshly prepared 0.1% SDS and 40 μl of chloroform to permeabilize the cells, 50 μl aliquots of the upper aqueous layer were transferred into 96-well flat-bottom microplates containing 150 μl of Z buffer and pre-equilibrated at 28 °C in the microplate reader. 40 μl of 0.4% ONPG were finally dispensed and the β-galactosidase reaction carried out at 28 °C for 40 min with measurement of the OD_420nm_ every 2 min. The relative β-galactosidase activity is calculated by the formula [(OD_420nm_ at time T2 − OD_420nm_ at time T1)/(T2 − T1)]/OD_600nm_. The T2 and T1 time points are chosen to be in the linear part of the kinetic.

### Construction of *M. smegmatis* strains expressing full-size and C-terminal truncated forms of MmpL3 fused to GFP

*Msmg*Δ*mmpL3*/pMVGH1-*mmpL3tb-gfp* and *Msmg*Δ*mmpL3*/pMVGH1-*mmpL3tb*^1–744^*-gfp* were generated as described previously^15^. Briefly, pMVGH1^4^ constructs expressing the *mmpL3* gene from *M*. *tuberculosis*, either full-size or truncated at its 3′-end so as to only express the first 744 amino acid residues of the protein, and fused to *gfp* and a C-terminal hexahistidine tag were used to transform a *M*. *smegmatis* strain having undergone a single crossover event at its *mmpL3* locus^4^. *Msmg*Δ*mmpL3* allelic exchange mutants expressing either *mmpL3tb*^1–744^*-gfp* or *mmpL3tb-gfp* were then selected by plating on 7H11-ADC agar containing Kan, Hyg and sucrose as described^15^.

### Fluorescence microscopy

*Msmg*Δ*mmpL3*/pMVGH1-*mmpL3tb-gfp* and *Msmg*Δ*mmpL3*/pMVGH1-*mmpL3tb*^1–744^*-gfp* cultures grown to exponential phase were collected, washed twice in phosphate-buffered saline containing 0.05% Tween 80, and fixed in freshly prepared 2% paraformaldehyde for 30 min at room temperature. Approximately 10^6^ cells were next transferred to a glass slide by Cytospin, mounted with Fluoro-Gel (Electron Microscopy Science) and visualized using a KEYENCE BZ-X700 fluorescence microscope. Fluorescent images for the two strains were acquired at identical exposure times. Multiple independent experiments were performed and data from one representative experiment were used for statistical analysis.

### Production and purification of CrgA, Rv0207c, LprC, Pks13, MviN and AftD

Recombinant forms of the CrgA, Rv0207c and Pks13 proteins from *M*. *tuberculosis* H37Rv harboring C-terminal hexahistidine tags were produced in *E*. *coli* Rosetta (DE3) using the pET29a (CrgA, Rv0207c) or pET26b (Pks13) expression systems (EMD Biosciences). AftD from *Mycobacterium abscessus* (1410 amino acids; 63% identity, 74% similarity to AftD from *M*. *tuberculosis* on a 1397 amino acid overlap) was expressed in *E*. *coli* BL21 (DE3) pLysS cells using a pNYCOMPS-N23 plasmid with a N-terminal 10-histidine tag. A recombinant form of LprC devoid of its first 21 amino acids (so as to remove the signal peptide of the protein including residue Cys21 predicted to be acylated) and harboring an N-terminal hexahistidine tag was produced in *E*. *coli* CR43 (DE3) using the pET14b expression system (EMD Biosciences). Similarly, *E*. *coli* C43 (DE3) cells harboring pET29a::MviN were utilized for purification of the His-tagged MviN. Overnight grown cells were sub-cultured 1:100 in fresh LB plus drug, and grown to OD_600_ 0.6–0.8, and then induced with 0.1 mM IPTG for four hours at 37 °C (CrgA, Pks13 and MviN), overnight at 16 °C (LprC and Rv0207c) or overnight at 22 °C (AftD). Cells were harvested by centrifugation at 4,000 × g for 20 min, then resuspended in lysis buffer (50 mM Tris pH 8.0, 5 mM EDTA, 1 mM PMSF) and broken by sonication. For AftD, the lysis buffer used was 20 mM HEPES pH 7.5, 200 mM NaCl, 20 mM MgSO_4_, 10 μg/mL DNase I (Roche), 8 μg/mL RNase A (Roche), 1 mM tris(2-carboxyethyl)phosphine hydrochloride (TCEP), 1 mM PMSF, 1 tablet/1.5 L buffer EDTA-free complete protease inhibitor cocktail (Roche). Unbroken cells were removed by centrifugation at 4,000 × g, and then membranes were collected by ultracentrifugation at 40,000 × g at 4 °C for 1 hour. Membranes were solubilized with HS buffer (20 mM HEPES pH 7.4, 150 mM NaCl, 2% Triton X-100) overnight at 4 °C with stirring. Insoluble material was removed by ultracentrifugation at 40,000 × g at 4 °C for 1 hour. Soluble samples were passed through a SP-HP column cation-exchange column (GE Healthcare) equilibrated in HS buffer, and flow-through was adjusted to 20 mM imidazole and 200 mM NaCl final concentration before loading onto HIS-bind column. The column was washed extensively with HW buffer (20 mM HEPES pH 7.4, 150 mM NaCl, 0.2% Triton X-100 supplemented with 20 mM and 50 mM imidazole; 60 mM imidazole for AftD), before elution (HW supplemented with 500 mM imidazole; 300 mM imidazole for AftD). Elution fractions were dialyzed against SPR Assay Buffer (20 mM HEPES pH 7.4, 150 mM NaCl, and 0.2% Triton X-100) and concentrated by ultrafiltration before SPR as necessary. 0.03% *n*-Dodecyl-β-D-maltoside (DDM), instead of 0.2% Triton X-100, was used in the SPR assay buffer in the case of AftD.

### Purification of MmpL3 and SPR experiments

*Msmg*Δ*mmpL3*/pMVGH1-*mmpL3tb* cells (expressing a C-terminally hexahistidine-tagged form of *mmpL3* from *M*. *tuberculosis* devoid of *gfp* fusion) were grown in four liters of 7H9-ADC medium supplemented with 0.2% glycerol, 0.05% Tween-80, 25 μg/mL kanamycin and 50 μg/mL hygromycin B for 48 hours at 30 °C with shaking. The MmpL3 protein was purified and SPR experiments were carried out as described previously^68^. The protein analytes in SPR assay buffer were injected over immobilized MmpL3 at 10 μL/min for 30 sec and then allowed to disassociate for 180 sec. Data was collected at 10 Hz and 25 °C. Similar results were obtained using Bio-layer interferometry (data not shown).

### Co-immunoprecipitation

*crgA* from *M*. *tuberculosis* H37Rv was cloned into pFAX, a mycobacterial integrative plasmid harboring an apramycin resistance cassette engineered in-house to allow for the expression of recombinant proteins N-terminally-fused to the FLAG epitope from the *hsp60* promoter. *M*. *smegmatis* Δ*mmpL3* cells co-expressing *mmpL3tb-gfp* from pMVGH1-*mmpL3tb-gfp* and *crgA-FLAG* from pFAX-*crgA* grown to an OD of ~0.8 were treated with 1.25 mM of the cross-linking agent dithiobis[succinimidyl propionate] (DSP; Thermo Scientific) at 37 °C for 30 min in PBS. Upon incubation with the cross-linker, bacteria were harvested, lysed by bead-beating in lysis buffer consisting of 50 mM Tris-HCl pH 7.5, 150 mM NaCl, 10 μg/ml DNAse I and proteinase inhibitor cocktail (Sigma-Aldrich), and the membranes collected by ultracentrifugation and resuspended in a buffer containing 50 mM Tris-HCl pH 7.5, 150 mM NaCl, 10% glycerol and 1% DDM (GoldBio). Upon solubilization of the membrane pellets overnight on ice, the insoluble material was removed by centrifugation at 27,000 × *g* at 4 °C for 30 min, and solubilized MmpL3tb and its binding partners purified on Ni-NTA agarose resin (ThermoFisher). Upon copious washing of the resin with washing buffer (50 mM Tris-HCl pH 8.0, 400 mM NaCl, 5% glycerol, 20 mM imidazole) to remove unbound proteins, cross-linked protein complexes were eluted from the column with elution buffer (50 mM Tris-HCl pH 8.0, 400 mM NaCl, 5% glycerol, 250 mM imidazole, 0.1% DDM). Elution fractions were run in 4–12% NuPAGE Bis-Tris Gels and the gels analyzed by in-gel fluorescence (to detect MmpL3tb-GFP) followed by Coomassie blue staining. DSP-induced complexes were reversed by incubation with dithiothreitol prior to loading on SDS-PAGE. Immunoblot analysis used anti-FLAG M2 antibodies (Sigma-Aldrich) as primary antibodies, and an anti-mouse IgG antibody coupled to horseradish peroxidase (HRP) (Sigma-Aldrich) as the secondary antibody. Radiance HRP substrate (Azure Biosystems) followed by chemiluminescence detection was used to detect the FLAG-tagged CrgA protein.

## Supplementary information

Supplementary Information
