# Supplementary material for: The MmpL3 interactome reveals a complex crosstalk between cell envelope biosynthesis and cell elongation and division in mycobacteria
Source: Sci Rep. 2019 Jul 24;9:10728. doi: 10.1038/s41598-019-47159-8 (PMC6656915; doi:10.1038/s41598-019-47159-8)

The MmpL3 interactome reveals a complex crosstalk between cell envelope biosynthesis and cell elongation and division in mycobacteria

Juan Manuel Belardinelli[1#], Casey M. Stevens[2#], Wei Li[1], Yong Zi Tan[3], Victoria Jones[1], Filippo Mancia[3], Helen I. Zgurskaya[2], Mary Jackson[1*]

[1]Mycobacteria Research Laboratories, Department of Microbiology, Immunology and Pathology, Colorado State University, Fort Collins, CO 80523-1682, USA; [2]University of Oklahoma, Department of Chemistry and Biochemistry, 101 Stephenson Parkway, Norman, OK 73019, USA; [3]Department of Physiology and Cellular Biophysics, Columbia University, 1150 St. Nicholas Avenue, New York, NY 10032, USA

<u>Table S1:</u> Kinetic parameters[a] of MmpL3 interactions with various proteins.

<u>Figure S1</u>: Quantification of *in vivo* interactions between full-size MmpL3 and enzymes involved in the biosynthesis of mycolic acids.

<u>Figure S2</u>: SDS-PAGE analysis of the partner proteins used in the SPR assay.

<u>Figure S3</u>: Quantification of *in vitro* interactions between MmpL3 and test proteins by surface plasmon resonance.

<u>Figure S4</u>: CrgA interaction with MmpL3 in intact mycobacterial cells.

Table S1. **Kinetic parameters[a] of MmpL3 interactions with various proteins.**

Ligand	k_a, M^{-1} s^{-1}	k_d, s^{-1}	K_D, μM	Residuals, χ^2
AftD	$(1.94 \pm 0.06) \times 10^4$	$(3.35 \pm 0.09) \times 10^{-3}$	0.17	894
CrgA	$(3.18 \pm 0.04) \times 10^3$	$(1.50 \pm 0.015) \times 10^{-3}$	0.47	25.1
Rv0207c	$(1.49 \pm 0.02) \times 10^4$	$(2.27 \pm 0.02) \times 10^{-3}$	0.15	24.6

[a] Data were fit globally using the 1:1 Langmuir binding model.

<u>Figure S1</u>: Quantification of *in vivo* interactions between full-size MmpL3 and enzymes involved in the biosynthesis of mycolic acids.

MmpL3 (full-size) proteins harboring C-terminal or N-terminal T18 and T25 domains were generated as “baits” and systematically compared for pairwise interactions with enzymes involved in the biosynthesis of mycolic acids [see Fig. 1]. Pairwise co-transformants (3 colonies of each) were grown in LB broth, and the cultures processed for β-galactosidase activity as described in Methods. The values presented are the mean activities (relative units) ± standard error from measurements performed on biological triplicates. The cut-off line was set at 5 times the average β-galactosidase activity measured in the negative control (T18/MmpL3^{T25N}).

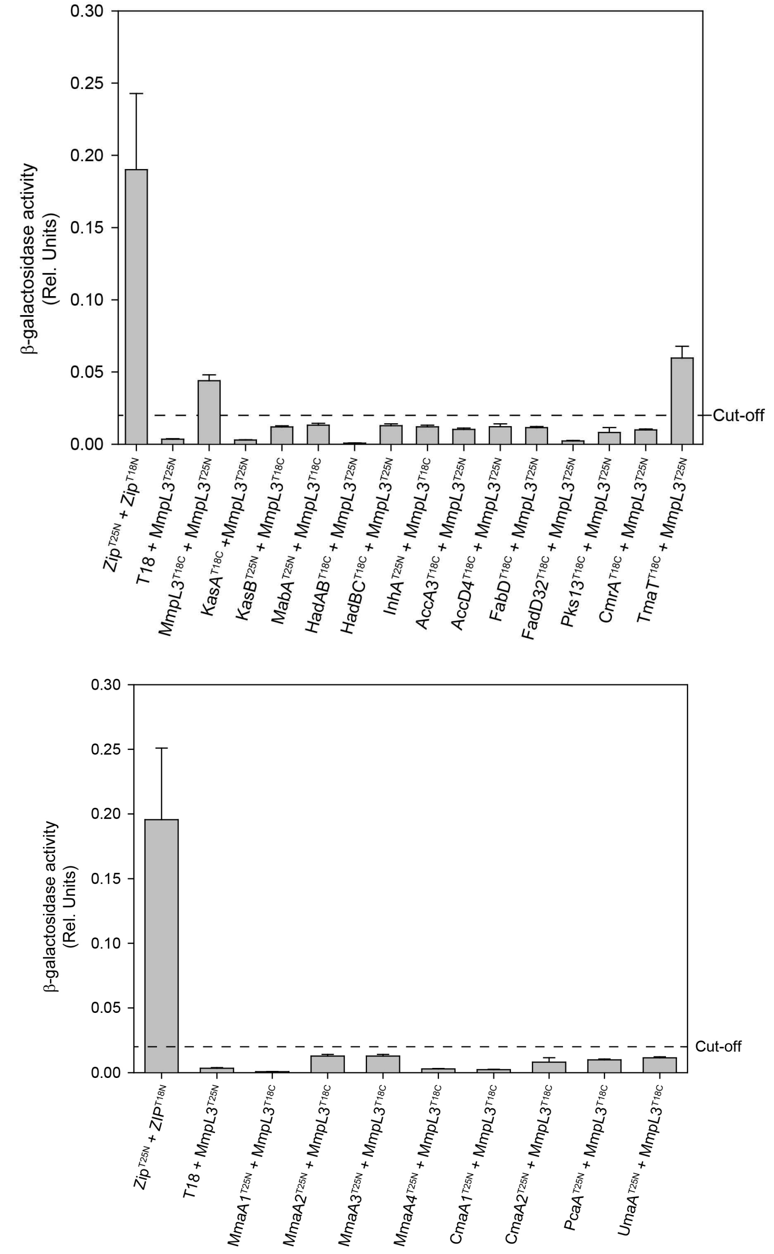

<u>Figure S2</u>: SDS-PAGE analysis of the partner proteins used in the SPR assay.
Proteins were purified as described in the Methods. Fractions eluted from the metal affinity purification step were analyzed by 8% (MmpL3), 16% (CrgA) and 12% (Rv0207c) SDS-PAGE. Purified and concentrated AftD was analyzed by 8% SDS-PAGE. All gels were stained with Coomassie Brilliant Blue. The low MW fragments in the MmpL3 sample constitute less than 3% of the total protein amount and are likely to be degradation products, as they co-purify with MmpL3 on a size exclusion column (data not shown).

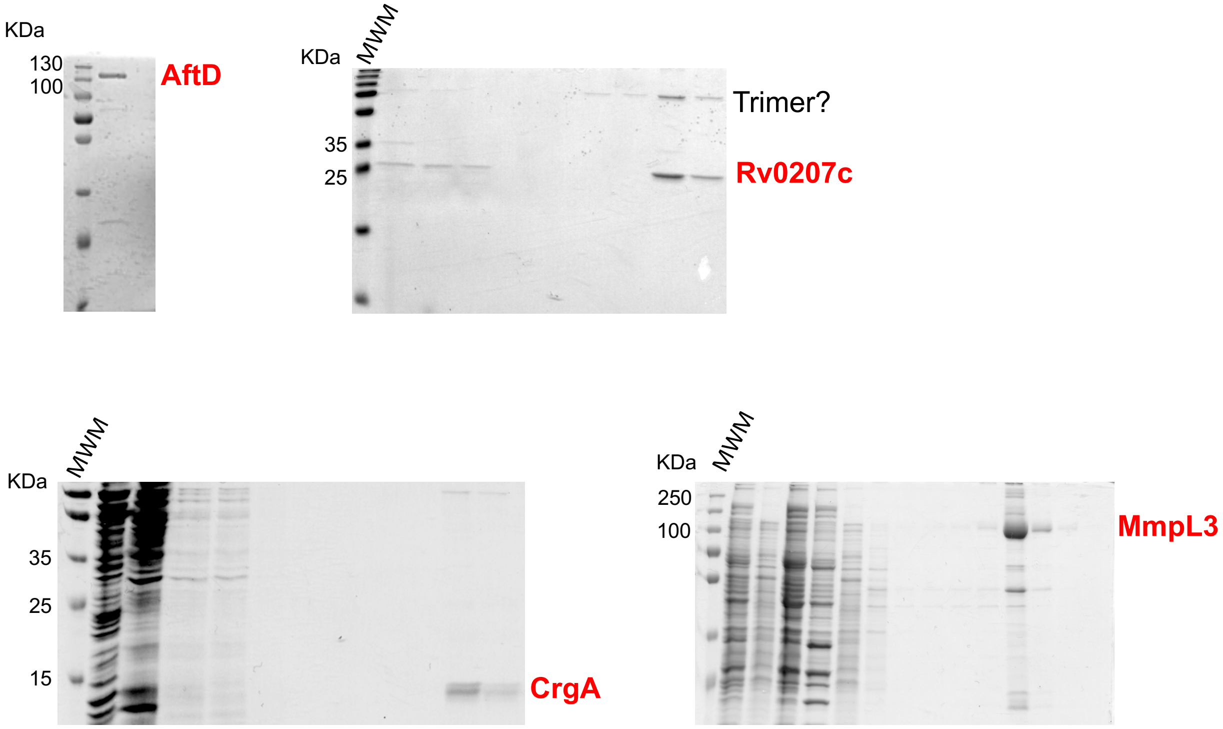

<u>Figure S3</u>: Quantification of *in vitro* interactions between MmpL3 and test proteins by surface plasmon resonance.
SPR was used to analyze kinetics of the purified MmpL3 interaction with various proteins. Binding sensorgrams were collected by injecting two-fold increasing concentrations ranging from 0.6 μM up to 10 μM of the indicated test proteins. No specific binding was detected for the mycolyltransferases FbpA, FbpB and FbpC (Ag85 complex; native purified proteins from BEI Resources), Pks13, MviN and LprC.

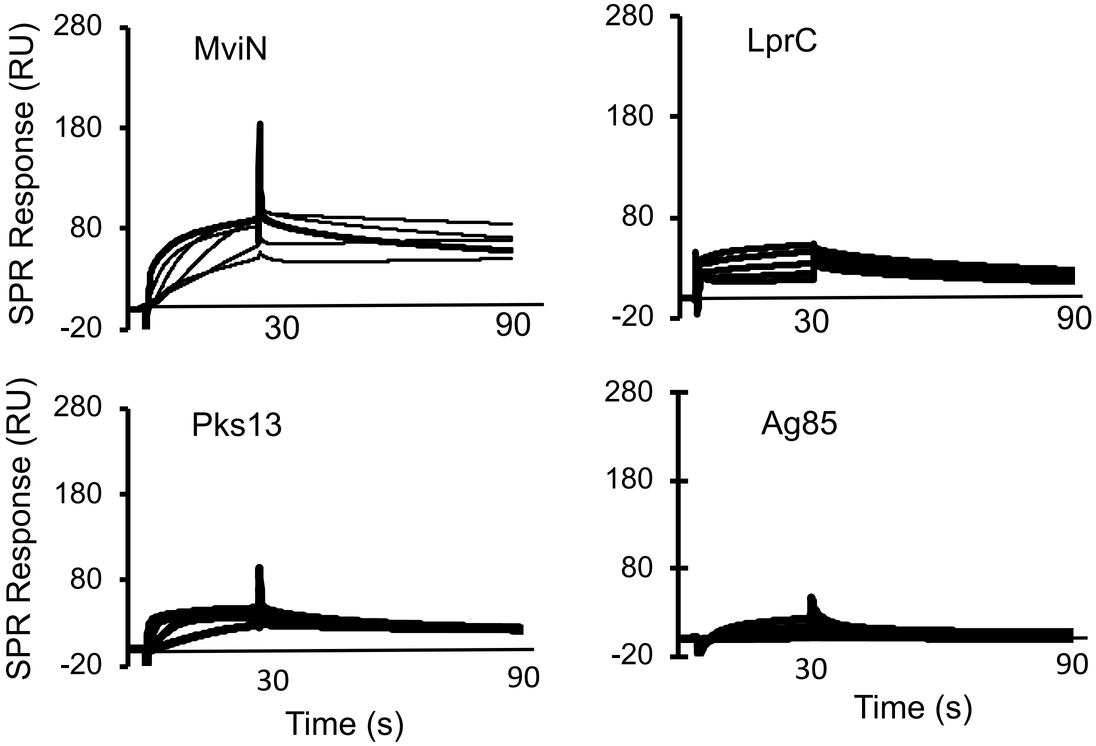

<u>Figure S4</u>: CrgA interaction with MmpL3 in intact mycobacterial cells.
In-gel fluorescence gels (right) and immunoblot analysis (left) of DSP-treated, detergent-solubilized, MmpL3tb-protein complexes prepared from *Msmg* + pFAX-*crgA*, *Msmg∆mmpL3*/pMVGH1-*mmpL3tb-gfp* + pFAX, and *Msmg∆mmpL3*/pMVGH1-*mmpL3tb-gfp* + pFAX-*crgA* cells.
In-gel fluorescence reveals the presence of high molecular weight MmpL3-GFP protein complexes in the elution fractions of strains expressing *mmpL3tb-gfp* that are reduced upon addition of DTT. The expected size of MmpL3-GFP is ~ 126 KDa. Immunoblots show the presence of CrgA-FLAG in the elution fractions from *Msmg∆mmpL3*/pMVGH1-*mmpL3tb-gfp* + pFAX-*crgA* cells but not in those from cells devoid of *mmpL3tb-gfp* or *crgA-FLAG* expression plasmids. The expected size of CrgA-FLAG is ~ 12 KDa. * denotes a non-specific *M. smegmatis* protein reacting with the anti-FLAG antibody. The same exposure was used for all blots. The full-length gel and blots are shown. Co-affinity purifications were performed twice on independent culture batches with the same results.

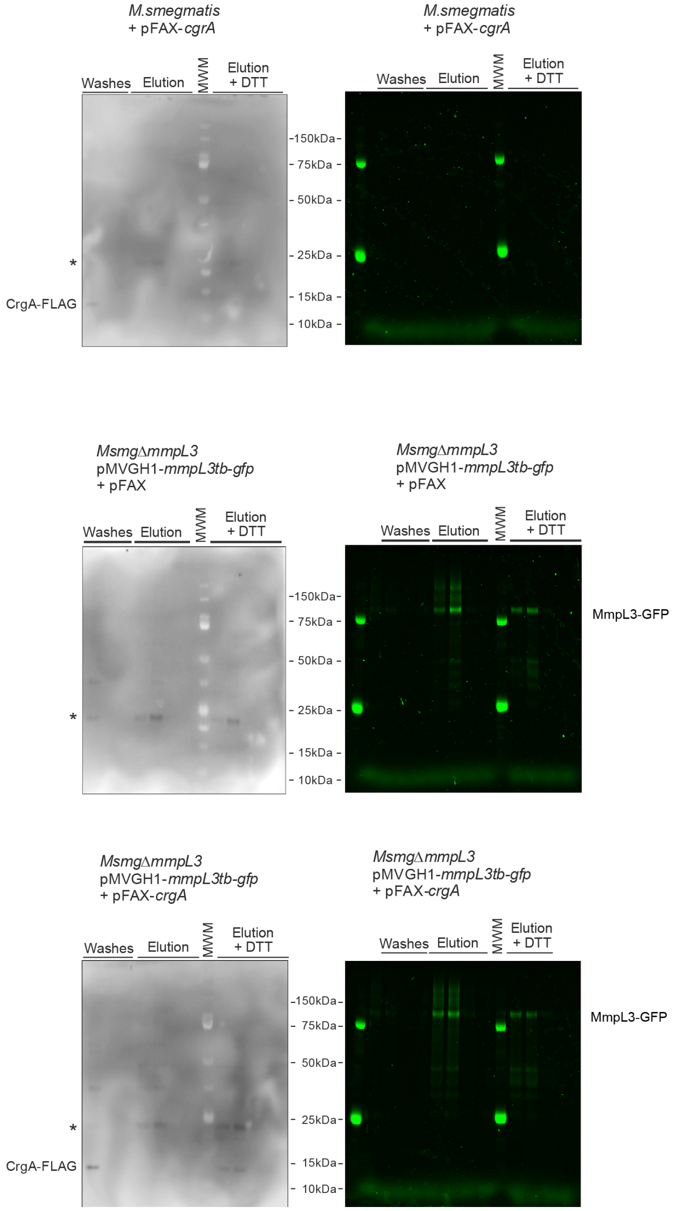

Supplement: Supplementary file 1 — Supplementary Information [file 41598_2019_47159_MOESM1_ESM.pdf]
